# The International Congress of Charities, Correction, and Philanthropy, June 12—18, 1893

**Published:** 1893-01-28

**Authors:** John S. Billings, Henry M. Hurd

**Affiliations:** Chairman; Secretary. The Johns Hopkins Hospital, Baltimore, U.S.A.


### THE WORLD'S FAIR.

### THE INTERNATIONAL CONGRESS OF CHARITIES, CORRECTION, AND PHILANTHROPY, JUNE 12—18, 1893.

SECTION FOUR ON THE HOSPITAL CARE OF THE SICK, THE TRAINING OF NURSES, DISPENSARY WORK, AND FIRST AID TO THE INJURED.

One of the series of International Congresses to be held in Chicago in 1893 is to be devoted to the subjects of Charities, Correction, and Philanthropy, and the Fourth Section of this is to consider all matters relating to the Hospital Care of the Sick, the Training of Nurses, Dispensary Work, and First Aid to the Injured. The Committee of Organisation of the Congress has appointed Dr. John S. Billings, Surgeon U.S. Army, as Chairman of this Section, and Dr. Henry M. Hurd, Superintendent of the Johns Hopkins Hospital in Baltimore, as its Secretary, and has authorized and requested them to complete its organisation, to extend invitations, and to prepare a programme for its work. Miss Isabel A. Hampton, Superintendent of the Training School for Nurses of the Johns Hopkins Hospital, has been appointed president of that part of the work of the Section which relates to the training of nurses. This Section will hold five sectional meetings of about two hours each, commencing June 12th, 1893, and will also have charge of one of the general sessions of the Congress, viz., that held on the morning of June 14th. It is desired that this shall be a truly international gathering for conference on the subjects alloted to this Section, and all who are interested in Hospitals, in Training of Nurses, in Dispensaries, or in First Aid to the Injured, are cordially invited to be present, to contribute papers, and to take part in the discussions. The papers and proceedings will probably be printed as a separate volume, and it is hoped that this will represent the best methods and the best work in each of these departments in all parts of the world. The following are suggested as subjects for special consideration in papers to be prepared: 1st, Hospital Organization—Governing Bodies—Relations of the Medical Staff and of Nurses' Training Schools; 2nd, Hospital Finances—Means of Support—Mode of Keeping Accounts—Cost; 3rd, Plan and Construction of recently-built General Hospitals, embodying the latest improvements; 4th, Relations of Hospitals to increase of Knowledge, to Medical Education, and to the Medical Profession—Hospital Records, Statistics, and Reports; 5th, Pay Patients in Hospitals; 6th, Isolating Wards and Hospitals for Contagious Diseases; 7th, Hospital Diets, Dietaries, Kitchens, &c.; 8th, Hospital Amphitheatres and Operating Rooms; 9th, Hospital Laundries and Disinfecting Establishments; 10th, Army and Navy Hospitals—Emergency Hospitals in Time of Epidemics—Temporary and Movable Hospitals; 11th, Small and Special Hospitals, Cottage Hospitals, School Hospitals, Private Hospitals, Sanatoriums, &c.—Convalescent Hospitals, and what to do with Incurables; 12th, History and present condition of Hospitals in the large cities; 13, Training Schools for Nurses (see special circular); 14th, Dispensaries—Relations to the Public and to the Medical Profession—Dispensary records; 15th, First Aid to the Injured—Associations for best means of popular instruction in and its place in General Education. Persons desiring to present papers, or to share in the discussions of this Section, are requested to communicate with the Secretary at once. The period of time allotted for the preparation of the programme is necessarily brief, and it is essential that all who are willing to assist in this work should act promptly.

JOHN S. BILLINGS, M.D., Chairman.
HENRY M. HURD, M.D., Secretary.

Address all communications to Dr. Henry M. Hurd, The Johns Hopkins Hospital, Baltimore, U.S.A.

